# Eating disorder symptomatology among transgender individuals: a systematic review and meta-analysis

**DOI:** 10.1186/s40337-023-00806-y

**Published:** 2023-05-26

**Authors:** Sofie M. Rasmussen, Martin K. Dalgaard, Mia Roloff, Mette Pinholt, Conni Skrubbeltrang, Loa Clausen, Gry Kjaersdam Telléus

**Affiliations:** 1 Psychiatry, Unit for Psychiatric Research, Aalborg University Hospital, Molleparkvej 10, 9000 Aalborg, Denmark; 2 Institute of Communication and Psychology, Psychology, Aalborg University, Aalborg, Denmark; 3 Department of Clinical Medicine, Faculty of Medicine, Aalborg University, Aalborg, Denmark; 4 Department of Child and Adolescent Psychiatry, Aarhus University Hospital, Aarhus, Denmark; 5 Department of Clinical Medicine, Faculty of Health, Aarhus University, Aarhus, Denmark; 6 Medical Library, Aalborg University Hospital, Aalborg, Denmark

**Keywords:** Eating disorder, Anorexia nervosa, Bulimia nervosa, Transgender, Gender identity, Gender-affirming treatment

## Abstract

**Objective:**

The purpose of this systematic review and meta-analysis was to synthesize the literature on eating disorders and eating disorder symptomatology among transgender individuals and to summarize the existing literature on gender-affirming treatment and the prevalence of eating disorder symptomatology.

**Method:**

The literature search for this systematic review and meta-analysis was performed in PubMed, Embase.com, and Ovid APA PsycInfo. We searched for “eating disorders” and “transgender” using both controlled vocabularies and natural language terms for their synonyms. The PRISMA statement guidelines were followed. Quantitative data from studies on transgender individuals and eating disorders assessed with relevant assessment tools was included.

**Results:**

Twenty-four studies were included for the qualitative synthesis, and 14 studies were included in the meta-analysis. The results revealed higher levels of eating disorder symptomatology among transgender individuals compared with cisgender individuals, especially cisgender men. Transgender men tend to display higher levels of eating disorder symptomatology than transgender women; however, transgender women seem to have higher levels of eating disorder symptomatology than cisgender men and, interestingly, this study also noted a trend toward transgender men having higher levels of eating disorders than cisgender women. Gender-affirming treatment seems to alleviate the presence of eating disorder symptomatology in transgender individuals.

**Discussion:**

The body of research on this subject is extremely limited, and transgender individuals are underrepresented in the eating disorder literature. More research investigating eating disorders and eating disorder symptomatology in transgender individuals and the relationship between gender-affirming treatment and eating disorder symptomatology is needed.

**Supplementary Information:**

The online version contains supplementary material available at 10.1186/s40337-023-00806-y.

## Abstract (highlights)

Research has found that transgender individuals are more likely to be diagnosed with an eating disorder or to engage in disordered eating than cisgender individuals. The purpose of this systematic review and meta-analysis was to synthesize the literature on eating disorders and eating disorder symptomatology among transgender individuals and to summarize the existing literature on gender-affirming treatment and the prevalence of eating disorder symptomatology. The literature search for this systematic review and meta-analysis was performed in online databases. Quantitative data from studies on transgender individuals and eating disorders assessed with relevant assessment tools was included. The results revealed higher levels of eating disorder symptomatology among transgender individuals compared with cisgender individuals, especially cisgender men. Gender-affirming treatment seems to alleviate the presence of eating disorder symptomatology in transgender individuals. The body of research on this subject is extremely limited, and transgender individuals are underrepresented in the eating disorder literature.

**Supplementary Information:**

The online version contains supplementary material available at 10.1186/s40337-023-00806-y.

## Background

Research has demonstrated that transgender individuals are more likely to be diagnosed with an eating disorder (ED) or to engage in disordered eating relative to cisgender individuals [1–4].Transgender is a term for individuals whose gender identity is different than what is typically associated with the sex-assigned-at-birth, such as transgender woman or transgender man [5]. Diemer et al. [1] investigated ED pathology and gender identity in a large population-based sample and found that transgender individuals had higher rate of past-year ED diagnosis, past-month use of diet pills, and purging or use of laxatives compared with cisgender women. Likewise, another study found higher rates of past-year ED among transgender individuals compared with cisgender women and men (17.6%, 1.8% and 0.2%, respectively) [2]. However, there are contradictory findings. Some researchers have suggested that EDs among transgender individuals is comparable to cisgender individuals or is rarely experienced [6–8]. Rabito-Alcón and Rodríguez-Molina [7] did not find any statistically significant difference in ED psychopathology between transgender individuals and comparison groups; however, transgender individuals presented with a higher level of body dissatisfaction (BD) compared with comparison groups. A systematic review by Jones et al. [9] investigated BD and disordered eating and found that the elevated levels of ED among transgender individuals may be related to the BD some transgender individuals experience in relation to the inconsistency between the sex-assigned-at-birth and the gender identity. In line with this, Ålgars et al. [10] found that the most frequent reason for EDs among transgender individuals was striving for thinness as way to suppress characteristics of the sex assigned at birth or to enhance characteristics of the gender identity. This was supported by several case studies in which the main reason for ED symptomatology was to control the physical shape to be more in line with the gender identity [11–13]. The research is sparse, though, and much of the literature is based on single-case studies or studies with small sample sizes [11–18].

Only a few studies have focused on the impact of gender-affirming treatment (GAT) (gender-affirming hormone treatment [GAHT], gender-affirming surgery [GAS], etc.) on the prevalence of ED symptomatology. Recent studies have shown that GAHT might alleviate ED symptoms, primarily through a positive impact on BD [19, 20]. BD is a core feature of ED psychopathology, and transgender individuals may be vulnerable to BD because of the distress and incongruence they may experience in relation to the body and gender [9, 21]. Thus, GAT may help alleviate ED symptoms or reduce the risk of developing EDs. Jones et al. [19] found that transgender individuals who were receiving GAHT reported statistically significant lower levels of ED psychopathology compared with individuals who were not receiving treatment. Likewise, in Ålgars et al.’s [10] study, the participants perceived GAT as alleviating symptoms of disordered eating. Still, case studies have found that some transgender individuals continued to experience ED symptoms after GAT [14, 16, 18]. Hence, the existing research that has investigated the impact of GAT in relation to ED psychopathology is conflicting, and a thorough review is needed. In this systematic review and meta-analysis, we aimed to systematically review the available literature on ED among transgender individuals and to review how GAT may impact ED symptoms in transgender individuals.

## Terminology

The language used respecting gender is somewhat inconsistent in the literature. In this paper, words like “female/woman” and “male/man” will be referred to as (presumed cisgender) woman and man. Further, the following terminology will be used throughout the paper: transgender men/males, transgender women/females, cisgender men/males, cisgender women/females. Control groups are referred to as comparison groups.

## Aim

This study aims to synthesize the literature on ED and ED symptomatology among transgender individuals through a systematic review of the literature. The study will also systematically review the literature on the relationship between GAT and the presence of ED symptomatology. Through meta-analyses, we examined differences in ED symptomatology between transgender and cisgender individuals as well as the prevalence of ED in transgender individuals.

To the best of our knowledge, this is the first meta-analysis on this topic ever conducted.

## Method

### Search strategy

The following databases were searched: PubMed, Embase.com, and Ovid APA PsycInfo. All databases were searched on February 28, 2022. The search strategy was developed by a medical librarian (CS) at The Medical Library, Aalborg University Hospital, Denmark, in cooperation with the other authors. We searched for terms related to “eating disorders” and “transgender” using both controlled vocabularies (i.e., MeSH terms) and natural language terms for their synonyms. The search was limited to articles in English, Danish, Norwegian, and Swedish. The search strategy was developed in PubMed and subsequently used in the other databases. A total of 1,202 unique citations were retrieved from the three databases. Duplicates were removed using Endnote and Rayyans [22] duplicate-identification strategies. The search strategies for all databases are listed in Additional file.

Furthermore, Rayyan [22] was used to screen articles and for full-text reading done by the raters independently.

### Inclusion and exclusion criteria

The PRISMA guidelines [23] were followed using a predeveloped inclusion and exclusion criteria guide in the systematic review and the meta-analysis.

To be included in the review, the studies had to be primarily quantitative studies with participants who identified as transgender or had been diagnosed with gender dysphoria according to the *International Classification of Diseases* (*ICD-10*; 24) or the *Diagnostic and Statistical Manual of Mental Disorders* (*DSM-IV* and *DSM-5*; 25, 26). Furthermore, the studies had to have assessed ED and/or ED symptomatology among the participants using a measurable instrument. Qualitative studies were included only if they reported quantitative scores of ED and/or ED symptomatology. In the case of studies with duplicated data sets, the review included the study with the highest number of transgender individuals with ED participating.

Case studies and studies with no clearly defined tool to measure ED were excluded. Studies also were excluded if they did not report on ED and/or ED symptomatology of transgender individuals, separately (e.g., if they reported on mixed groups of gender-diverse individuals). Studies in languages other than English, Danish, Norwegian, or Swedish were excluded as well, as were handbooks, theses, manuals, conference notes and posters.

### Eating disorders measures

The following paragraph lists the assessment tools used in the articles included in this paper.

The Eating Disorder Examination Questionnaire (EDE-Q; 27), which assesses ED behavior and weight concerns within the past 28 days. The EDE-Q is a self-report questionnaire consisting of 28 items distributed in four subscales and a global score. The questions are based on a 7-point Likert scale. A higher score on EDE-Q means a higher degree of ED pathology.

The Eating Disorder Examination Questionnaire Short (EDE-QS) is a 12-item version of the EDE-Q based on a 4-point Likert-scale ranging from 0 to 3. The EDE-QS is measuring ED symptoms similarly to the EDE-Q. A higher score indicates greater ED pathology [28].

The Eating Attitudes Test (EAT) is a self-report questionnaire that assesses symptoms of eating pathology. The EAT exists in both a shorter version, the EAT-26 (26 items), and the original version, the EAT-40 (40 items) [29, 30]. The questions are rated on a 6-point Likert scale that ranges from 1 (*always*) to 6 (*never*). A higher score on the EAT-26 and the EAT-40 means a higher degree of ED pathology.

The Eating Disorder Inventory (EDI) is a self-report questionnaire that identifies risk of developing an ED or pathology as well as documenting an ED. The questions are rated on a 6-point Likert scale that ranges from *never* to *always*. There are three versions of the EDI: the EDI-1 [31] 64 items in eight subscales), EDI-2 ([32]; 91 items in 11 subscales), and the EDI-3 ([33]; 91 items in 12 subscales).

The Eating Pathology Symptoms Inventory (EPSI; [34]) is a 45-item self-report measure that assesses ED psychopathology. The EPSI includes eight subscales and is scored on a 5-point Likert scale that ranges from *never* to *very often.* A higher score indicates higher levels of ED psychopathology.

The Sick, Control, One stone, Fat, Food Questionnaire (SCOFF; [35]) is a five-item screening tool that assesses whether an ED is present or not. Scores range from 0 to 5. A score ≥ 2 indicates a potential ED.

The Mini-International Neuropsychiatric Interview (M.I.N.I.; [36]) is a short structured diagnostic interview that assesses psychiatric diagnoses from *DSM*-*4* and *ICD*-*10*.

The Diagnostic Interview Schedule for Children (DISC; [37]) assesses psychiatric diagnoses among children and adolescents. It is a computerized, structured interview.

The Structured Clinical Interview for DSM-IV Axis I Disorders (*SCID I*; [38]) is a screening tool for psychiatric diagnoses.

Studies were included in the meta-analyses if they reported either ED measures for both trans- and cisgender populations or prevalence of ED for transgender populations using the following assessment instruments; EDE-Q, EDI, EAT, EPSI, and SCOFF. All instruments are recognized ED diagnostic or screening instruments aimed at measuring ED symptoms. Studies were excluded from the meta-analyses if the studies did not use a recognized ED instrument for measuring ED symptoms, as mentioned above, for either trans- and cisgender populations or prevalence of ED for transgender populations.

### Statistics

Three meta-analyses were performed depending on the type of reported results and included populations. The first meta-analysis included all studies reporting different ED measures for trans- and cisgender populations and used the standardized mean difference between these populations as the summary statistic. The second meta-analysis included the subgroup of the studies from the first meta-analysis that reported the EDE-Q and used the mean difference between these populations as the summary statistic. The third meta-analysis included all studies that reported prevalence of ED for transgender populations assessed by the previously mentioned ED-specific questionnaires.

For the first and second meta-analyses, we converted the scores from studies that reported only the mean and standard deviation of an instrument’s subscales into estimates of the global score (which is usually calculated as the mean of the subscales) such that they could be compared with the global scores reported by the other studies. The mean of the global score was computed as the mean of the reported subscales’ means. The variance of the global score requires the covariances between the subscales in order to be computed precisely (see Olofsson and Andersson [39], p. 199). Because these covariances were unknown, lower and upper bounds on the variance of the global score were formed by assuming that the covariances were 0 and by using the Cauchy–Schwarz inequality (see Keener [40], p. 71), respectively.

For the third meta-analysis, the variance can possibly be squeezed toward 0 for low or high prevalences, which means that the study would be given an unreasonably large weight when using the inverse variance method. Therefore, as suggested in Barendregt et al. [41], prevalences close to 0 or 100% should be transformed through the Freeman–Tukey double-arcsine transform, and the confidence should then be derived via back-transformation by using the inverse of the pooled variance.

The heterogeneities in each meta-analysis were evaluated on the basis of the ${I}^{2}$ statistic. Random-effects models with the DerSimonian–Laird method were generally used in the main analyses as significant heterogeneities were found, and fixed-effects models were used in sensitivity analyses. For the first and second meta-analyses, the primary analyses were made with the upper bound on the variance (because it gives a smaller weight to the studies affected), and the lower bound was used in another sensitivity analysis.

In all meta-analyses, possible outliers were detected using the DFFITS and COVRATIO diagnostics as suggested in Viechtbauer and Cheung [42]. Both are computed for each study and examine the influence of it by comparing the model fitting when the study is removed. The DFFITS diagnostic expresses the standardized difference between the predicted average effects, and its absolute value will therefore be relatively high for outliers. On the other hand, the COVRATIO diagnostic expresses the ratio of the variances without and with each study, which means that a value below 1 indicates that a more precise estimate can be obtained by excluding that study.

The analyses were performed in Stata (Version 16; [43]) with the METAN package (Version 4.05 by David Fisher) for making forest plots and META/METAPRED (the latter by Ariel Linden) for outlier diagnostics.

### Quality assessment

All articles were reviewed independently and blinded for each other by three of the authors (SMR, MR and MP) and subsequently consensus rated to ensure the quality of the selection process. Disagreements were remedied through discussion until a consensus was reached. The quality of the included studies was assessed using the Joanna Briggs Institute (JBI) Critical Appraisal Checklist for Analytical Cross-Sectional studies, Quasi-Experimental Studies, and Qualitative Research. The three tools evaluate different items in the studies, which was answered “Yes”, “No”, “Unclear” or “Not Applicable”. The JBI checklist for Analytical Cross-Sectional studies are rated on a scale ranging from 1 to 8, the checklist for Quasi-Experimental studies ranges from 1 to 9 and the checklist for Qualitative Research ranges from 1 to 10. The JBI provides a total summed score for an overall appraisal to include or exclude the studies. The total summed scores are provided in Table 1.

**Table 1:** Qualitative synthesis

References	Country	Group (n)	Population	Mean age (SD)	Eating disorder measures	Mean (SD) EDE-Q global score	Transition status	Results	Quality
Arikawa et al. [53]	USA	Transgender men (59) Male (presumed cisgender) (29) Female (presumed cisgender) (93)	Community sample recruited from universities and local businesses		EAT-26 EDE-Q	2.2 (1.5) 1.8 (1.0) 2.3 (1.3)	GAHT (81,8%)	Transgender men had higher levels of ED than (presumed cisgender) males	5
Cella et al. [45]	Italy	Transgender women (15)	Community sample recruited from associations and universities	44.60	EDI-2 DSM-VI		None had GAS	Transgender women reported higher levels of ED than cisgender individuals	5
Duffy et al. [49]	USA	Transgender women (19) Transgender men (22)	Community sample recruited from community organizations and transgender organizations		EDE-QS			Transgender women had higher ED scores than transgender men	5
Gómez-Gil et al. [56]	Spain	Transgender women (159) Transgender men (71)	Clinical sample of patients with gender dysphoria complaints recruited at a hospital		M.I.N.I		All had applyed for GAHT or GAS GAHT (104) transgender women and (10) transgender men	Low prevalence of ED	5
Hepp et al. [57]	Switzerland	Transgender women (20) Transgender men (11)	Clinical sample of outpatients undergoing treatment for GAS	33.2 (10.3)^1^	SCID-I DSM-VI		GAHT (10) GAS (7)	Low prevalence of ED	3
Jones et al. [18]	UK	Transgender individuals (563)	Clinical sample recruited from national transgender health service	29.49 (13.67)	EDI-2		GAHT (139) Not in GAHT (416)	Transgender individuals in GAHT had significantly lower ED symptoms than individuals not in GAHT	5
Khoosal et al. [6]	UK	Transgender women (40)	Clinical sample from gender identity clinic	41.8	EDI-2		All transgender women received GAS and GAHT	Transgender women did not report higher levels of ED before or after GAS compared to comparison group	6^a^
Linsenmeyer et al. [52]	USA	Transgender men (128) Transgender women (28)	Clinical sample recruited from a gender identity clinic		SCOFF		The majority were in GAHT (78.4%)	28% of the sample screened positive for an ED	5
Lipson et al. [58]	USA	Transgender individuals (330) Cisgender individuals (63,994)	Community sample based on students recruited from institutions		SCOFF			Transgender individuals had significantly higher ED scores compared to cisgender individuals	5
Mitchell et al. [51]	USA	Transgender men (42) Transgender women (41)	Community sample recruited from community websites, blogs and snowball sampling	28.26 (9.9) 36.68 (18.1)	EDE-Q (Restraint subscala)	1.55 (1.5) 1.95 (1.9)		Misgendering was associated with decreased dietary restraint in transgender men	5
Mustanski et al. [8]	USA	Transgender women (12) Transgender men (8) Male (presumed cisgender) (107) Female (presumed cisgender) (119)	Community sample recruited via multiple methods and living in the Chicargo area	18.31(1.32)^2^	DISC DSM-VI			No prevalence of ED in transgender individuals	5
Nowaskie et al. [54]	USA	Transgender men (79) Transgender women (87)	Clinical sample recruited from an outpatient gender health program	27.18 (10.19) 34.69 (14.34)	EDE-Q	1.30 (1.11) 1.62 (1.28)	GAHT (84) GAHT and GAS (30)	Transgender women and transgender men had EDs above cutoff, 13.8% and 10.1%, respectively Transgender individuals who had recieved GAHT and GAS had lower levels of ED than transgender individuals not in GAHT	5
Nagata et al. [43]	USA	Transgender men (312) Transgender women (172)	Community sample recruited from a national cohort study collected through a web-based platform	30.5 (9.7), 41.2 (14.9)	EDE-Q	1.76 (1.36) 1.83 (1.28)		Transgender individuals had significantly higher rates of ED compared to cisgender individuals	5
Peterson et al. [44]	USA	Transgender women (69) Transgender men (180)	Clinical sample recruited from a gender identity clinic	17.04 (2.88)	EDE-Q	1.63 (1.40) 1.61 (1.33)	GAHT (28%)	Transgender individuals had significantly higher ED scores compared to cisgender individuals	5
Rabito-Alcón et al. [7]	Spain	Transgender individuals (61) Comparison group (40)	Clinical sample recruited from a gender identity clinic	27.28 (6.60) 21.85 (2.24)	EAT-26 EDI-2 (Body dissattisfaction subscale)		All in assessment phase at gender identity clinic	No significant difference between transgender individuals and the comparison group	5
Roberts et al. [50]	USA	Transgender male (635) Transgender female (64) Cisgender male (231) Cisgender female (688)	Community sample recruited from social media through advertisement	16.0 (1.2) 16.2 (1.2) 15.9 (1.1) 15.8 (1.1)	EPSI			Transgender individuals had significantly higher rates of ED compared to cisgender individuals	5
Romano and Lipson et al. [55]	USA	Transgender men (679) Transgender women (278)	Community sample recruited from student populations at institutions	21.49 (4.99) 22.28 (5.96)	SCOFF			32.57% of the transgender men and 34.23% of the transgender women reported positive for an ED on the SCOFF	5
Schvey et al. [48]	USA	Transgender males (95) Transgender females (87)	Community sample recruited through ilitary installation and social media	36.84 (15.29) 30.17 (11.27)	EDE-Q	0.97 (1.0) 2.2 (1.5)	The majority were in GAT	Transgender individuals had higher ED scores than cisgender men	5
Testa et al. [19]	USA	Transgender women (154) Transgender men (288)	Community sample recruited online through advertisments directed at transgender organisations	24.59 (4.90) 22.70 (3.50)	EAT-26		Transgender individuals who either wanted or had accessed GAT	23% of the transgender women and 22% of the transgender men reported ED GAT was associated with decreased ED symptoms	5
Turan et al. [46]	Turkey	Transgender women (37) Comparison group of (presumed cisgender) women (40)	Clinical sample recruited from a hospital based on individual who had applied for GAT	24.59 (4.90) 22.70 (3.50)	EAT-40		All transgender men were in GAHT	No significant difference between transgender men and the comparison group and no significant difference before/after GAHT	8^a^
Vocks et al. [47]	Germany	Transgender women (88) Transgender men (43) Comparison group of (presumed cisgender) men (56) and women (107)	Participants were recruited from self-help groups, counseling center and gender identity clinics	37.27 (11.18) 34.95 (7.99) 34.77 (12.91) 32.80 (13.22)	EDE-Q EDI-2	1.82 (0.71) 1.63 (0.69)	GAHT: 57% of transgender women and 61% of trangender men GAS: 18% of transgender women and 33% of trangender men	Transgender individuals showed significantly higher ED scores compared to the comparison group No significant correlations were found for the number of transition stages	4
Witcomb et al. [20]	UK	Transgender individuals (200) Comparison group of cisgender individuals (200)	Clinical sampel recruited from a gender identity clinic	29.45 (6.70) 35.20 (12.10)	EDI-2		GAHT 15 (10.7%)	Transgender individuals scored significantly higher on BD than cisgender individuals	4
Ålgars et al. [10]	Finland	Transgender men (11) Transgender women (9)	Community sample recruited from transgender support service	42.22 (13.82) 29.45 (6.70)	EDI-3		All but 4 participants had undergone or were currently undergoing GAT	ED scores were higher than in samples of (presumed cisgender) males	7^b^
Ålgars et al. [4]	Finland	Transgender individuals (571) Comparison group (571)	Population based sample recruited from the Finnish registry	25.15 (4.47)^3^	EAT-26			Transgender men showed sigificantly higher levels of ED symptoms than the comparison group of (presumed cisgender) females	4

*GAT* gender-affirming treatment, *GAHT* gender-affirming hormone treatment, *GAS* gender-affirming surgery. The quality was assessed with JBI Critical Appraisal Checklist for: Analytical Cross-Sectional Studies, ranging on a scale from 1 to 8, ^a^Quasi-Experimental Studies ranging on a scale from 1 to 9 and ^b^Qualitative Research ranging on a scale from 1 to 10. ^1^Including both transgender men and women. ^2^Including both transgender individuals, (presumed cisgender) males and females. ^3^Including both transgender individuals and comparison groups

## Results

### Article selection

**Fig. 1:**
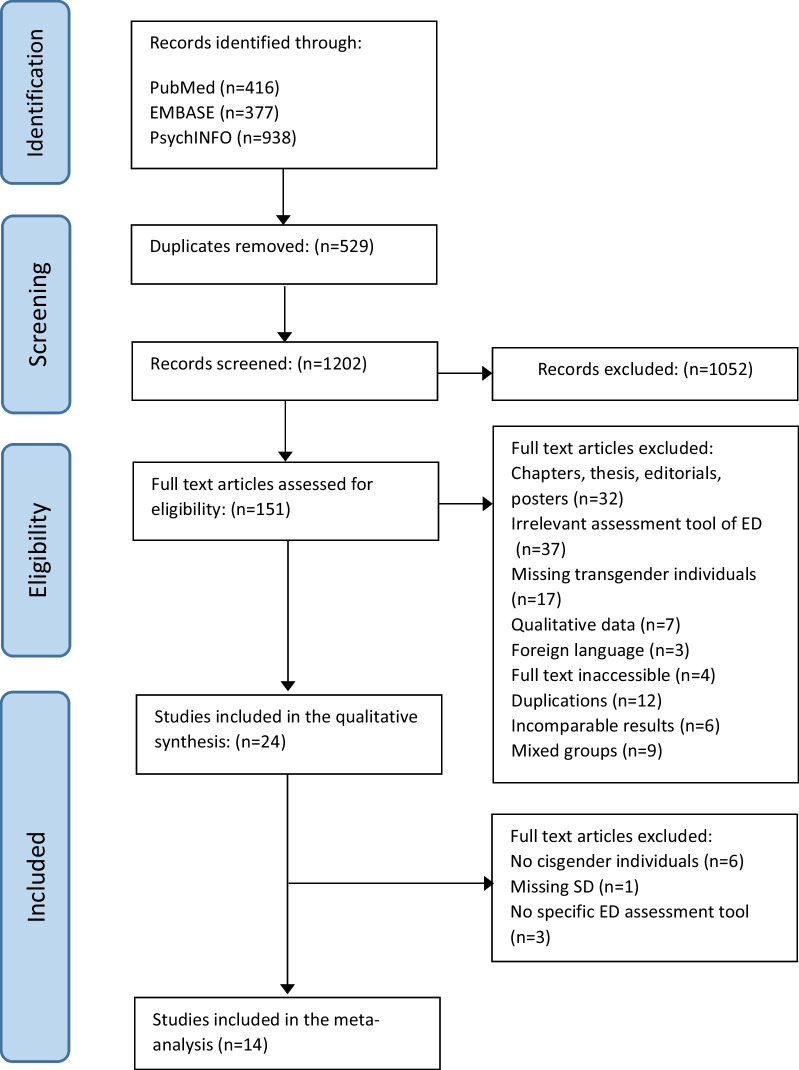
Flow diagram. Results of literature search with exclusion criteria as well as the numbers of studies included and excluded

The literature search resulted in 1202 studies and the subsequent screening resulted in 24 studies included in the systematic review and 14 studies in the meta-analysis. The flow diagram in Fig. 1 presents a detailed description of the screening process including reasons for exclusion.

### Qualitative synthesis

Table 1 shows the data extracted from the included studies in the systematic review on author, publication year, country, sample size, population, eating disorder measure, diagnostic system of ED, mean age (SD) EDE-Q global score, transition status, and results. Information about mean age and SD were reported when possible. Exclusion criteria were described, as were the numbers of studies included and excluded.

A total of 24 studies were included in this systematic review. Twenty studies specifically explored EDs and ED symptomatology among transgender individuals [4, 6, 7, 10, 19–21, 44–56], while four studies investigated mental health and psychiatric disorders, including ED psychopathology among transgender individuals [8, 57–59]. Of the four studies, three reported DSM-IV diagnoses, while one reported degree of symptoms. Six of the articles explored the relationship between GAT and ED among transgender individuals [6, 19, 20, 47, 48, 55]. The majority of the studies were cross-sectional, 2 were characterized as pre–post studies, and 1 was a qualitative study. For further descriptive information about the studies, see Table 1.

Fifteen articles found high rates of ED symptomatology among transgender individuals using ED-specific assessment tools such as the EDE-Q, EDI, EAT, EPSI, and SCOFF [4, 10, 20, 21, 44–46, 48, 49, 51, 53–56, 59]. The majority of these studies found higher ED scores in transgender individuals compared with cisgender individuals [4, 10, 21, 44–46, 48, 49, 51, 54, 59]. Nagata et al. [44] found high EDE-Q scores, especially with regard to shape concern, in both transgender men and women compared with comparison groups, with transgender men scoring higher on measures of eating concern, weight concern, shape concern, and global scores compared with age-matched (presumed cisgender) men, and transgender women had higher scores on weight concern, shape concern, and the global score compared with age-matched (presumed cisgender) women. Another study that used only a single EDE-Q subscale (Restraint) found that misgendering was associated with increased dietary restraint in transgender men and increased BD in transgender women [52]. Peterson et al. [45] found that transgender individuals had significantly higher total scores on the EDE-Q compared with existing samples of undergraduate cisgender men and a sample of cisgender men and women. They found high rates of objective binge eating in transgender men and women: 23.4% the past month and 3.9% self-induced vomiting in the past month. In Ålgars et al.’s [10] study, 70% of the transgender individuals in the study reported past or current disordered eating, with the mean EDI-3 scores higher than those of (presumed cisgender) men, whereas, compared with existing samples of (presumed cisgender) women, scores were only slightly higher on Bulimia. However, none of the participants reached clinical levels of ED. A recent study by Roberts et al. [51] found that transgender males exhibited significantly higher purging compared with cisgender males, more calorie restriction than cisgender females, and higher muscle building than cisgender men and women. Transgender females exhibited significantly higher calorie restriction than cisgender males. They displayed significantly less muscle building, and excessive exercise, compared with cisgender male and females. Witcomb et al. [21] found that transgender women and men had higher levels of ED symptomatology compared with cisgender comparison groups, but not as high as those of patients with ED. Vocks et al. [48] found that transgender women reported a higher level of disturbed eating and body image disturbance than did comparison groups of (presumed cisgender) males and females. They found that transgender men did only differ from (presumed cisgender) male comparison groups. Finally, both transgender men and women showed lower degrees of ED and BD than (presumed cisgender) women with ED. Cella et al. [46] reported that transgender women were at high risk of ED, with transgender women reporting higher levels of ED compared with cisgender individuals. Similar results were reported by Schvey et al. [49] who found that transgender women reported statistically significant higher ED symptomatology according to EDE-Q compared with transgender men. Furthermore, they found that transgender individuals exhibited more ED symptomatology than existing community samples of (presumed cisgender) men. Ålgars et al. [4] found that transgender men had significantly higher ED scores compared with comparison groups of (presumed cisgender) females, but for transgender women no difference was found compared with comparison groups of (presumed cisgender) males. Furthermore, they reported that transgender men had higher levels of disordered eating and BD than did transgender women on all items and scale scores of the EAT-26 [4].

Six articles reported ED scores above the clinical cutoff range among transgender individuals, with the prevalence of ED ranging from 13.8 to 34.23% in the samples [20, 53–56, 59]. Romano and Lipson [56] found the highest prevalence of ED in a sample of transgender individuals, with transgender women having the highest prevalence of ED. Another study found that 28% of the sample screened positive for an ED on the SCOFF. It is important to mention that this finding included nonbinary individuals in the sample; however, the study reported mean and *SD* scores on the SCOFF of transgender and nonbinary groups separately [53]. High rates of ED were found in Testa et al.’s [20] study; 23% of transgender women and 22% of transgender men exhibited ED scores above cutoff on the EAT-26. Another study found that 20% of the transgender men were at risk or met the criteria for an ED compared with 6.9% of cisgender men and 16.1% of cisgender women according to the EAT-26 [54]. This study did also find that transgender men had higher proportions of ED, as assessed by the EDE-Q, compared with cisgender men, but not cisgender women [54]. Lipson et al. [59] showed a prevalence of ED among transgender students that was almost two times higher than that of cisgender students, 13.17% and 8.37%, respectively [59]. Last, Nowaskie et al. [55] found that 13.8% of transgender women and 10.1% of transgender men had a probable clinical ED based on the cutoff of the EDE-Q.

Three articles found low levels of ED scores among transgender individuals, and a further three articles found lower ED scores among transgender individuals compared with comparison groups [6–8, 47, 57, 60]. Gómez-Gil et al. [57], Hepp et al. [60] and Mustanski et al. [8] all conducted clinical interviews and found low or no prevalence of ED in their samples of transgender individuals using global assessment tools of psychopathology (such as SCID, M.I.N.I. and DISC). Turan et al. [47], Khoosal et al. [6] and Rabito-Alcón and Rodríguez-Molina [7] all investigated ED in transgender individuals compared with comparison groups and did not find that transgender individuals reported higher ED scores compared with comparison groups. Still, all three studies found that transgender individuals presented with high levels of BD [6, 7, 47].

Six articles explored the relationship between GAT and ED among transgender individuals [6, 19, 20, 47, 48, 55]. Despite mixed results, based on larger studies by Testa et al. [20], Jones et al. [19] and Nowaskie et al. [55] there seem to be a tendency toward GAT having an alleviating impact on ED symptoms. Jones et al. [19] found that transgender individuals who were not receiving GAHT reported higher levels of ED psychopathology than individuals who were receiving GAHT. They also found that GAHT alleviated ED symptoms primarily through a positive impact on BD and that it reduced perfectionism, anxiety symptoms, and increased self-esteem. Testa et al. [20] found that GAT was indirectly associated with fewer ED symptoms for transgender men and transgender women, via a pathway from less nonaffirmation (e.g., when other people use the wrong name or pronouns) of gender identity to lower levels of BD. Nowaskie et al. [55] found that transgender individuals who had received both GAHT and gender-affirming surgery (GAS) reported lower ED symptomatology than participants who had received only GAHT and participants who had not received any treatment. However, transgender individuals who had received only GAHT did not report lower ED symptoms compared with participants who had not received either GAHT or GAS. In contrast, studies by Turan et al. [47], Khoosal et al. [6] and Vocks et al. [48] did not find that GAT alleviated ED symptoms. Despite this, Turan et al. [47] and Khoosal et al. [6] found that BD decreased significantly after treatment in transgender individuals, which is similar to the findings noted by Jones et al. [19] and Testa et al. [20]. Overall, the majority of the studies suggested that transgender individuals seem to display higher ED symptomatology compared with cisgender individuals and that GAT somewhat seems to have a positive impact on ED symptomatology.

### Meta-analyses

The primary objective of this study was to examine differences in ED symptomatology between transgender and cisgender individuals. Of the 24 studies included in the systematic review (see Fig. 1), a total of 14 studies were included in the meta-analyses while 10 studies were excluded (the reasons for excluding are shown in the Additional file).

**Fig. 2:**
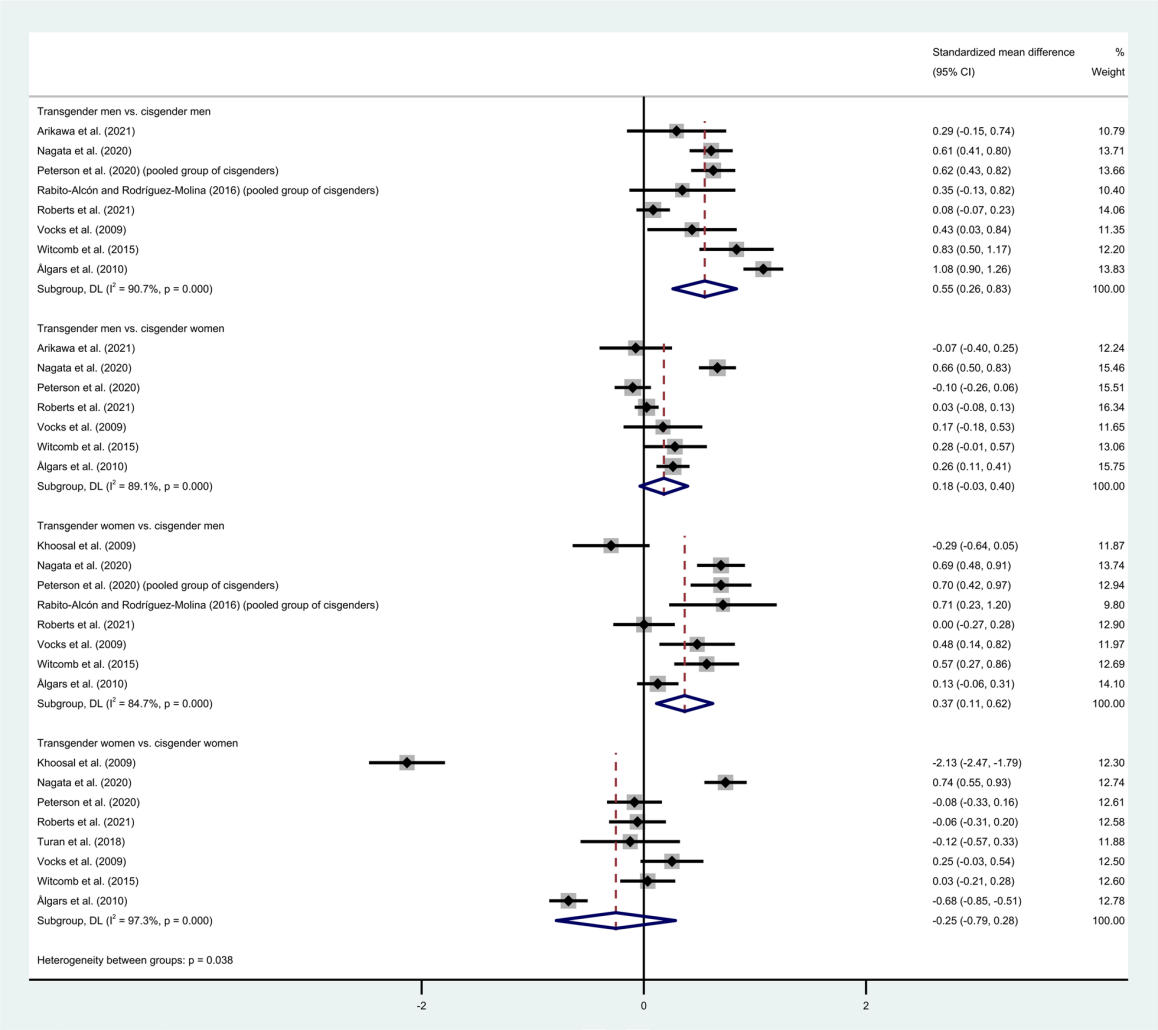
Meta-analysis on eating disorder symptomatology in transgender men and women versus. cisgender men and women. Forest plots on transgender men and transgender women versus cisgender men and women. The pooled estimates have been computed with a random-effects model because of the levels of heterogeneity. The global scores for Khoosal et al. [6], Roberts et al. [51], Vocks et al. [48], and Witcomb et al. [21] were computed with the upper bound for the variance given that only subscales are reported. Note that Rabito-Alcón and Rodríguez-Molina [7] and Peterson et al. [45] reported results only for pooled groups of cisgender individuals. The instruments used in these studies are the EAT-26, EAT-40, EDE-Q, EDI (with the same three subscales), and EPSI

Studies that used various instruments to assess ED symptomatology were included in the forest plots shown in Fig. 2. Random-effects models were used to compute the pooled estimates for each of the groups, giving pooled standardized mean differences (with 95% CIs) of 0.55 (0.26, 0.83) and 0.18 (− 0.03, 0.40) for transgender men compared with cisgender men and women, respectively, and of − 0.25 (− 0.79, 0.28) and 0.37 (0.11, 0.62) for transgender women compared with cisgender women and men, respectively, with considerable levels of heterogeneity (${I}^{2}\ge 84.7\%)$ found in all four comparisons ([61]; see Sect. 9.5.3). For the studies that reported only subscales, the upper bound on the variance of the global score was used. Note that only the subscales Body Dissatisfaction, Bulimia, and Drive for Thinness were used for the studies that reported EDI scores because these studies did not report scores for all subscales. Sensitivity analyses with combinations of random-/fixed-effects models and the upper/lower bounds on the variance were conducted, which showed results similar to those presented in Fig. 2. Furthermore, various studies were identified as possible outliers and excluded in an additional sensitivity analysis, which also showed results similar to those presented in Fig. 2. See the additional materials for results on the sensitivity analyses and which studies were identified as possible outliers.

**Fig. 3:**
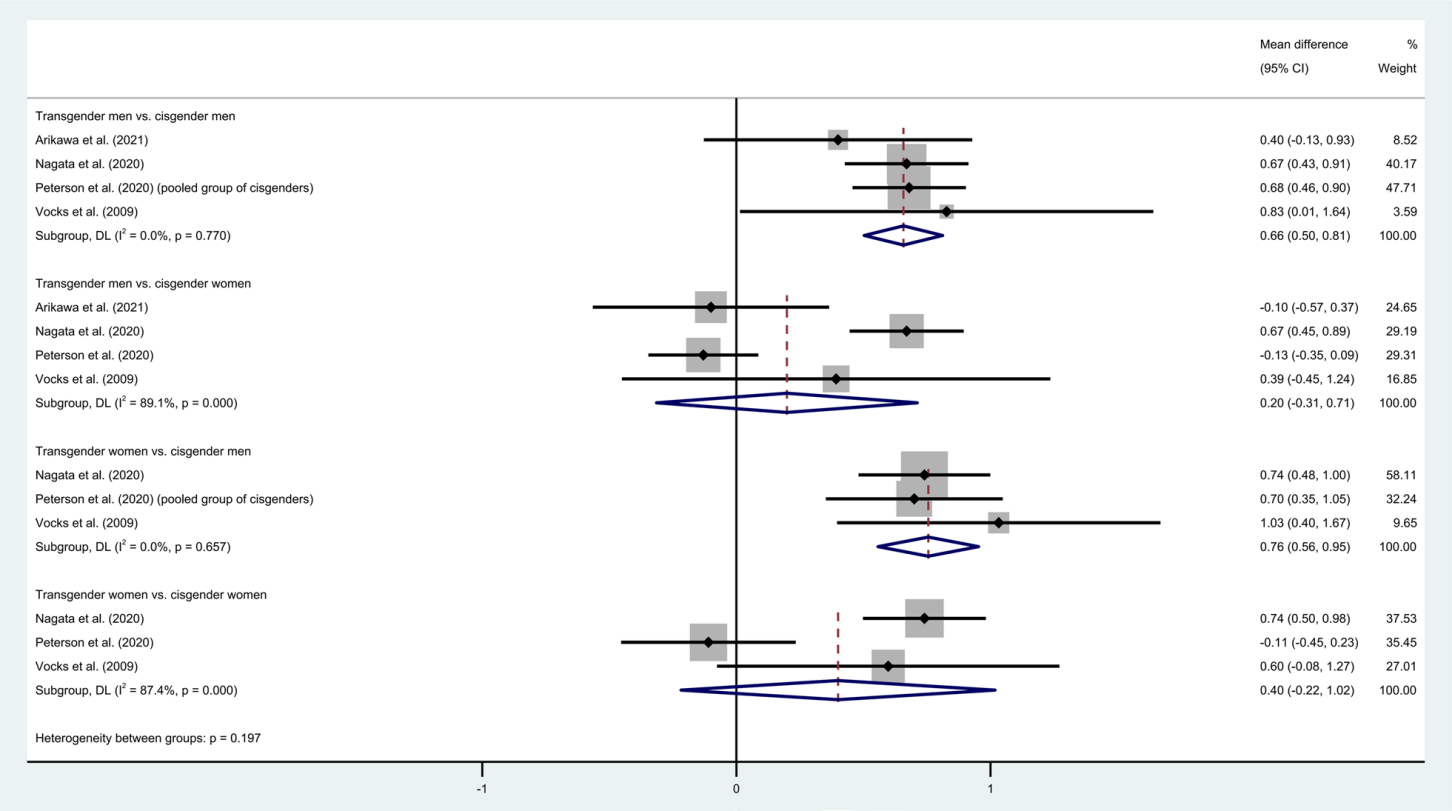
Meta-analysis on Eating Disorder Examination Questionnaire (EDE-Q) in transgender men and women versus cisgender men and women. Legend: Forest plots on transgender men and women vs. cisgender men and cisgender women with only the studies that reported EDE-Q scores. The pooled estimates have been computed with a random-effects model (because the fixed-effects and random-effects models are equivalent in the case of no heterogeneity). The global scores for Vocks et al. [48] were computed with the upper bound for the variance because only subscales are reported. Note that Peterson et al. [45] reported results only for pooled groups of cisgender individuals. The meta-analysis was conducted based on EDE-Q as the EDE-Q has a 28-day time frame which enables the instrument to identify the frequency of key ED behaviors opposite to other self-report instruments of ED (e.g. EAT and EDI; 30, 32)

Figure 3 shows forest plots of studies using only the EDE-Q, finding pooled mean differences (with 95% CIs) of 0.66 (0.50, 0.81) and 0.20 (− 0.31, 0.71) for transgender men compared with cisgender men and women, respectively, and of 0.40 (− 0.22, 1.02) and 0.76 (0.56, 0.95) for transgender women compared with cisgender women and men, respectively. The comparisons with cisgender men are both statistically significant and have levels of heterogeneity of 0.0%, while the comparisons with cisgender women have considerable levels of heterogeneity (${I}^{2}\ge 92.8\%)$. The upper bound on the variance of the global score was used for Vocks et al.’s [48] study, in which only the subscales were reported. Similarly, to the first meta-analysis, various sensitivity analyses were made, which showed results similar to those in Fig. 3. However, due to the low number of studies and because outliers could not be clearly identified in all comparisons, the study by Nagata et al. [44] was removed as a possible outlier in the comparison between transgender men and cisgender women.

**Fig. 4:**
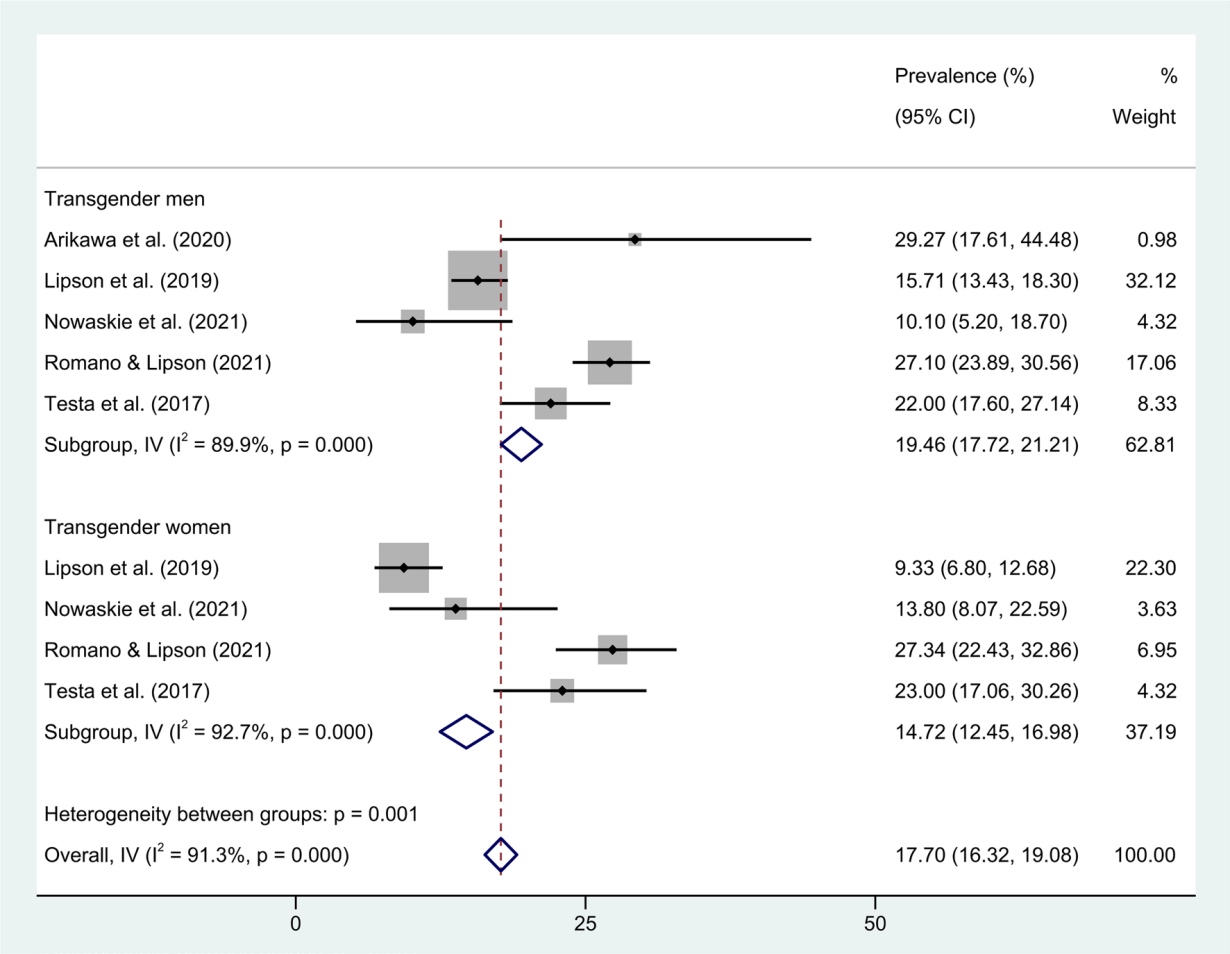
Meta-analysis on the prevalence of eating disorders in transgender individuals. Forest plots on the prevalences of eating disorders in transgender men and women. The pooled estimates have been computed with a fixed-effects model because of the considerable sizes of the studies by Lipson et al. [59] and Romano and Lipson [56], which would have smaller weights in a random-effects model. The instruments used in the studies were the Eating Attitudes Test (26-item version), SCOFF, and Eating Disorders Examination Questionnaire

Figure 4 shows a forest plot of studies that reported the prevalence of EDs in transgender men and women (based only on ED-specific instruments), finding pooled prevalences in percentages (with 95% CIs) of 19.46 (17.72, 21.21) and 14.72 (12.45, 16.98) for transgender men and women, respectively, and 17.70 (16.32, 19.08) overall. Despite considerable levels of heterogeneity (${I}^{2}\ge 89.9\%$) being found, fixed-effects models were used in the main analysis. The reason for this is that the studies by Romano and Lipson [56] and Lipson et al. [59] are rather large, but random-effects models (which one would typically use in case of considerable levels of heterogeneity) would give these studies weights comparable to the other studies. A random-effects model was used in a sensitivity analysis, which gave slightly larger prevalences but also much wider confidence intervals. The Freeman–Tukey double-arcsine transform previously mentioned was not used because the prevalences reported by the included studies were not close to 0. The outlier diagnostics (see Additional file) do not give a clear picture of which studies are possible outliers, but the studies by Nowaskie et al. [55] and Lipson et al. [59] generally report rather low prevalences of ED compared to the studies by Arikawa et al. [54], Romano and Lipson [56] and Testa et al. [20], which report higher prevalences of ED. Therefore, two sensitivity analyses with these groups of studies were made (see Additional file), which found significantly lower and higher prevalences of ED compared to the results shown in Fig. 4, respectively.

## Discussion

To the best of our knowledge, this is the first meta-analysis to compare ED symptomatology between transgender and cisgender individuals and to estimate the prevalence of ED in transgender individuals, and to explore the relationship between GAT and ED psychopathology in transgender individuals.

The results from the qualitative synthesis showed that transgender individuals generally display higher levels of ED and ED symptomatology compared with cisgender individuals. This finding was supported by the meta-analysis, which also indicated that especially transgender men display higher levels of ED symptomatology compared to transgender women.

Overall, the studies from the qualitative synthesis that have found higher rates of ED symptomatology included larger numbers of transgender participants, and they used ED-specific assessment tools, as opposed to the studies that found lower or no prevalence of ED. For these studies methodological issues are important to consider. First, these studies in general included small sample sizes, ranging from 20 to 61 transgender individuals [6–8, 47, 60] with only one study by Gómez-Gil et al. [57] including a higher participant rate (*n* = 230). Second, three studies used clinical diagnostic interviews to investigate ED psychopathology using global assessment tools of psychopathology (such as the SCID I, M.I.N.I., and DISC; 8, 57, 60). All the studies which assessed ED applying a global clinical interview found low prevalence of ED among transgender individuals in contrast to ED-specific assessment. Furthermore, the results from the qualitative synthesis are in accordance with the results of a previous systematic review that found transgender individuals to be engaging in disordered eating and that BD may increase the risk of disordered eating for some transgender individuals [9].

The first meta-analysis of this paper found significant differences on ED pathology between both transgender men/women and cisgender men. Furthermore, tendencies of transgender men to score higher than cisgender women were seen, but these results were not significant. Considerable levels of heterogeneity were found for all subgroups, which make the results less reliable. This could possibly be due to the different types of ED instruments used in the studies, which make it more difficult to make comparisons across the studies.

When only the EDE-Q scores were included in the second meta-analysis, significant differences were found between both transgender men and women versus cisgender men (see Fig. 3). Interestingly, the meta-analysis found that transgender women have higher levels of ED symptomatology than cisgender men, although they are both assigned male sex at birth. From a clinical perspective, it is important to mention that even though we found that transgender men and women scored significantly higher on measures of ED than cisgender men, they did not score above the clinical cutoff on the EDE-Q (see Table 1). Considerable levels of heterogeneity were also found in this analysis for the comparisons with cisgender women, which make these results less reliable. When comparing transgender men to cisgender women and transgender women to cisgender men, it is potentially problematic from a gender equity perspective as this implies that the assigned sex at birth drives risk of ED. However, simply reproducing the comparisons made in the original studies, may not be the most useful way to summarizing the literature, as this could also lead to misleading conclusions. This exemplifies the complexity of this research field and calls for more research from the ED literature discussing the usefulness and appropriateness of what comparisons are being conducted. Furthermore, for solid conclusions, more studies using EDE-Q in transgender populations are needed in future research.

The third meta-analysis estimated the overall prevalence of EDs in transgender individuals to be 17.70% (see Fig. 4), which is significantly higher compared with the general population. Results from a previous meta-analysis have estimated a lifetime prevalence of ED at 1.01% (95% CI (0.54, 1.89)) in the general population [62]. Furthermore, on the basis of the fixed-effects model from the meta-analysis there is very strong evidence that the prevalences of ED for transgender men are significantly higher than for transgender women (see Fig. 4). However, considerable levels of heterogeneity were also found in this analysis. Among others, these levels of heterogeneity may be due to the levels of cut-off used in the studies. In a study by Romano and Lipson [56], who found rather high prevalences of ED in transgender populations, is used a cut-off for SCOFF of ≥ 2, while Lipson et al. [59], who found lower prevalences of ED in transgender populations, is used a cut-off for SCOFF ≥ 3. Therefore, these studies are not directly comparable, which is why sensitivity analyses with studies reporting low and high prevalences of ED were made.

The meta-analysis points to a potentially increased level of ED symptomatology in transgender men compared with transgender women. A possible explanation for this could be that transgender men strive for thinness to suppress characteristics of the assigned sex, such as breast, hips, and menstruation [10, 18, 63]. The difference in gender identity is also evident in a clinical population of people with ED, with (presumed cisgender) females being at higher risk of ED compared with (presumed cisgender) males [64–66]. Researchers have estimated that anorexia nervosa has a heritability ranging from 56 to 74% in (presumed cisgender) females, which suggests that genetic factors significantly influence the risk for this disorder [67–70]. In combination with the drive for suppressing characteristics of the assigned sex, this could be a contributing factor as to why transgender men tend to show higher levels of ED symptomatology than transgender women. Overall, when considering the findings of this paper it is important to mention that it is not ultimately the sex-assigned-at-birth that impacts ED related outcomes [71, 72]. Many transgender individuals experience harassment, discrimination and internalized transphobia and all factors have been associated with eating related psychopathology among transgender individuals [71–74]. Thus, this perspective may contribute to help explain why transgender individuals experience higher levels of ED compared to cisgender individuals.

When we explored the impact of GAT on ED and ED symptomatology in transgender individuals, we found somewhat mixed results. However, large studies by Testa et al. [20], Jones et al. [19] and Nowaskie et al. [55] have found a tendency pointing towards GAT having an alleviating impact on ED symptoms in transgender individuals. These studies provide some of the most specific evidence recently conducted and include larger samples of transgender individuals, compared with the studies of Turan et al. [47], Khoosal et al. [6] and Vocks et al. [48], which included lower numbers of transgender participants (see Table 1). The finding of this paper suggests that BD may play a somewhat important role regarding ED and that GAT may be effective interventions to improve BD and thereby alleviating ED symptomatology [19, 20]. In Ålgars et al. [10] several of the participants described having experienced reductions in ED symptoms and improved body image after GAT. Likewise, in Jones et al.’s [9] systematic review, which investigated BD and disordered eating in transgender individuals, the results also indicated that GAT alleviated BD. However, not all transgender individuals will receive GAT and, therefore, GAT will not be a uniform protective factor for all transgender individuals. Protective factors such as connectedness to family and school, having friends and receiving social support have been linked to lower odds of ED behaviors among transgender youth [71]. Thus, it is important to pay attention to how other factors than GAT may be protective or alleviating of ED.

A general issue when investigating the relationship between GAT and ED is how the onset and continuation of GAT may affect ED symptomatology in transgender individuals. Only Turan et al. [47] and Khoosal et al. [6] conducted pre-post studies with assessment of ED before and after the GAT within the same participants (follow-up approximately after 6 months in both studies). However, the studies investigated different treatment interventions which is restricting our ability to compare the studies. Khoosal et al. [6] investigated GAS (but with participants who had been prescribed with GAHT for an average period of 32 month) and Turan et al. [47] investigated GAHT. Moreover, both studies only included transgender women and low numbers of participants (*n* = 37 and *n* = 40). The rest of the studies are cross-sectional studies which are comparing transgender individuals “who have received GAT” to transgender individuals “who have not received GAT” and the group of individuals “who have received GAT” include a mix of individuals at various stages of transition at the time for assessment in the studies [19, 20, 48, 55]. Transgender individuals who are further along in GAT may experience more congruence with the gender identity and subsequently improved ED, whereas transgender individuals who are in the beginning of GAT may experience less congruence with the gender identity [55]. This issue could be a possible reason why this paper has found less clear results, which in turn restricted our ability to draw general conclusions about the relationship between GAT and the presence of ED symptomatology. Furthermore, several of these studies were cross-sectional and thus could not determine causality. Unfortunately, it was not possible to conduct a meta-analysis regarding the relationship between GAT and ED symptomatology because of the limited literature available on this subject.

## Strengths and limitations

There are several strengths to this review that should be mentioned. First, all the studies were reviewed by three authors, which ensured the quality of the selection process. Second, to ensure the quality of this review, we accepted only measurable instruments as appropriate for reporting ED. However, there are some limitations that should be mentioned. The existing literature has several limitations, which have an important impact on this review and analysis. First, the existing literature is generally based on case studies and studies with small sample sizes. Because of the rather low number of studies used in the meta-analyses, it was not possible to (a) use funnel plots to examine for publication bias and small study effects or to (b) use meta-regression to adjust for covariates and explore the reasons for the variety found between the included studies’ results [61].

Second, the lack of consistency in measures used to assess ED tends to make comparisons across the studies more difficult (e.g., the M.I.N.I., SCID I, and DISC are all assessment tools of general psychopathology and therefore they may not provide a comprehensive understanding of ED as assessment tools of ED). When assessing EDs, ED-specific interviews such as Eating Disorder Examination [75], are preferred to self-reported questionnaires for a comprehensive assessment of ED. However, based on the findings from this study, it seems that ED-specific self-reporting questionnaires are preferrable for capturing ED when compared to interviews based on global instruments of psychopathology in transgender individuals. Even though ED-specific interviews are preferable, it is not always possible to conduct interviews in larger studies and therefore ED-specific self-reported questionnaire are preferred to assess EDs. Furthermore, it is important to mention that even though this paper found high levels of ED among transgender individuals, only a few of the included studies mentioned the use of diagnostic system of ED, such as DSM or ICD, and therefore could not determine final diagnosis of ED among transgender individuals. See Table 1 as to specifications of diagnostic systems used in the articles.

Third, the populations included in this review were recruited through various sampling methods and consisted of both clinical and community samples. Only a few studies have included clinical samples; most have used community samples of transgender individuals recruited from various organizations, social media platforms, and so on. Therefore, the transgender individuals included in the studies may not be representative of the transgender population as a whole. Also, comparison groups differed regarding gender; for example, some studies compared transgender men with cisgender males, and other studies compared them with cisgender females. This lack of consistency makes it more difficult to make comparisons across the studies and restricts our ability to draw general conclusions. Still, it is important to mention that the findings of this review are consistent with those of the previous systematic review by Jones et al. [9]. Furthermore, we did not include articles that studied nonbinary individuals. Thus, future research should aim to investigate EDs among nonbinary individuals because this group is even more underrepresented in the research field of ED.

## Conclusion

This study is, to the best of our knowledge, the first meta-analysis to investigate eating disorder psychopathology between transgender individuals and cisgender individuals and the first systematic review since 2016. It is also the first to systematically review the available literature regarding the relationship between gender-affirming treatment and eating disorder psychopathology in transgender individuals. Our results indicate that transgender individuals present with higher levels of eating disorder symptomatology relative to cisgender individuals, especially relative to cisgender men. Also, the results indicate that transgender men tend to have higher levels of eating disorders than transgender women. However, the results showed that transgender women seem to present with higher rates of eating disorder symptomatology compared with cisgender men and, interestingly, this study also found tendencies of transgender men to have a higher levels of eating disorders than cisgender women. Moreover, the results indicate that gender-affirming treatment seems to be somewhat alleviating of eating disorder symptomatology.

## Supplementary Information

**Additional file 1.** Title: Search protocol. Description: Search strategies for databases.

**Additional file 2.** Title: Reasons for excluding studies from the meta-analyses. Description: Reasons for excluding studies from the meta-analyses.

**Additional file 3.** Title: Data used for meta-analyses of mean differences. Description: Data used for meta-analyses of mean differences.

**Additional file 4.** Title: Data used for meta-analysis of prevalence. Description: Data used for meta-analysis of prevalence.

**Additional file 5.** Title: Results from all meta-analyses. Description: Results from all meta-analyses.

**Additional file 6.** Title: Outlier diagnostics from the meta-analyses. Description: Outlier diagnostics from the meta-analyses.

## Data Availability

The datasets generated and/or analysed during the current study are not publicly available due to this paper being a review article.
