# Supplementary material for: Eating disorder symptomatology among transgender individuals: a systematic review and meta-analysis
Source: J Eat Disord. 2023 May 26;11:84. doi: 10.1186/s40337-023-00806-y (PMC10214585; doi:10.1186/s40337-023-00806-y)
Supplement: Supplementary file 1 — Additional file 1. Title: Search protocol. Description: Search strategies for databases. [file 40337_2023_806_MOESM1_ESM.docx]

## Search protocol

Date 22.08.2022

## PubMed

| Search number | Search Details | Results |
| --- | --- | --- |
| 3 | ("Sexual and Gender Disorders"[MeSH Terms] OR "Transgender Persons"[MeSH Terms] OR "Transsexualism"[MeSH Terms] OR "Gender Dysphoria"[MeSH Terms] OR "gender nonconform*"[Text Word] OR "gender non conform*"[Text Word] OR "gender variant*"[Text Word] OR "gender incongru*"[Text Word] OR "non binar*"[Text Word] OR "nonbinar*"[Text Word] OR "transgender*"[Text Word] OR "transsexual*"[Text Word] OR "gender dysphor*"[Text Word] OR "trans person*"[Text Word] OR "trans people*"[Text Word] OR "transpeople*"[Text Word] OR "trans sexual*"[Text Word] OR "sexual dysphor*"[Text Word] OR "gender disorder*"[Text Word] OR "gender identit*"[Text Word]) AND ("Feeding and Eating Disorders"[MeSH Terms] OR "disordered eating*"[Text Word] OR "anorex*"[Text Word] OR "bulimia*"[Text Word] OR "binge eating*"[Text Word] OR "OSFED"[Text Word] OR "EDNOS"[Text Word] OR "eating disorder*"[Text Word] OR "overeating"[Text Word]) AND ("english"[Language] OR "danish"[Language] OR "swedish"[Language] OR "norwegian"[Language]) | 416 |

## Embase

| No. | Query | Results |
| --- | --- | --- |
| #12 | #10 AND #11 | 377 |
| #11 | danish:la OR english:la OR swedish:la OR norwegian:la | 34650742 |
| #10 | #5 AND #9 | 403 |
| #9 | #6 OR #7 OR #8 | 99921 |
| #8 | anorex*:ti,ab,kw OR bulimia:ti,ab,kw OR 'binge eat*':ti,ab,kw OR osfed:ti,ab,kw OR ednos:ti,ab,kw OR overeat*:ti,ab,kw | 66055 |
| #7 | (eating NEAR/2 disorder*):ti,ab,kw | 32497 |
| #6 | 'eating disorder'/exp | 59967 |
| #5 | #1 OR #2 OR #3 OR #4 | 23033 |
| #4 | transpeople*:ti,ab,kw OR transsexual*:ti,ab,kw OR transgender*:ti,ab,kw OR 'non binar':ti,ab,kw OR nonbinar*:ti,ab,kw OR 'sexual dysphor*':ti,ab,kw | 14946 |
| #3 | (trans NEAR/2 (person* OR people* OR sexual*)):ti,ab,kw | 589 |
| #2 | (gender* NEAR/2 (disorder* OR dysphor* OR nonconform* OR 'non conform*' OR incongru* OR variant* OR identit*)):ti,ab,kw | 9532 |
| #1 | 'transgender'/exp OR 'transsexuality'/exp OR 'gender dysphoria'/exp | 15228 |

## PsycInfo

| **#** | **Query** | **Results** |
| --- | --- | --- |
| 1 | exp eating disorders/ | 33,138 |
| 2 | (anorex* or bulimia or 'binge eat*' or osfed or ednos or overeat*).mp. | 30,708 |
| 3 | (eating adj2 disorder*).mp. | 34,658 |
| 4 | 1 or 2 or 3 | 49,700 |
| 5 | gender dysphoria/ | 1,123 |
| 6 | exp gender identity/ | 42,462 |
| 7 | (transpeople* or transsexual* or transgender* or 'non binar' or nonbinar* or 'sexual dysphor*').mp. | 13,594 |
| 8 | (trans adj2 (person* or people* or sexual*)).mp. | 634 |
| 9 | (gender* adj2 (disorder* or dysphor* or nonconform* or 'non conform*' or incongru* or variant* or identit*)).mp. | 26,348 |
| 10 | 5 or 6 or 7 or 8 or 9 | 57,192 |
| 11 | 4 and 10 | 1,004 |
| 12 | limit 11 to (danish or english or norwegian or swedish) | 938 |
