# Supplementary material for: Eating disorder symptomatology among transgender individuals: a systematic review and meta-analysis
Source: J Eat Disord. 2023 May 26;11:84. doi: 10.1186/s40337-023-00806-y (PMC10214585; doi:10.1186/s40337-023-00806-y)
Supplement: Supplementary file 2 — Additional file 2. Title: Reasons for excluding studies from the meta-analyses. Description: Reasons for excluding studies from the meta-analyses. [file 40337_2023_806_MOESM2_ESM.pdf]

**Author (year)**	**Type of statistic reported**	**Excluded due to**
Jones et al. (2017)	ED symptomatology	Not reporting scores on cisgenders
Ålgars et al. (2012)	ED symptomatology	Not reporting scores on cisgenders
Duffy et al. (2021)	ED symptomatology	Not reporting scores on cisgenders
Linsenmeyer et al. (2021)	ED symptomatology	Not reporting scores on cisgenders
Schvey et al. (2020)	ED symptomatology	Not reporting scores on cisgenders
Mitchell et al. (2012)	ED symptomatology	Not reporting scores on cisgenders
Cella et al. (2013)	ED symptomatology	Not reporting standard deviations
Hepp et al. (2005)	Prevalence of ED	Not ED-specific assessment tool
Mustanski et al. (2010)	Prevalence of ED	Not ED-specific assessment tool
Gómez-Gil et al. (2009)	Prevalence of ED	Not ED-specific assessment tool
