# Supplementary material for: Eating disorder symptomatology among transgender individuals: a systematic review and meta-analysis
Source: J Eat Disord. 2023 May 26;11:84. doi: 10.1186/s40337-023-00806-y (PMC10214585; doi:10.1186/s40337-023-00806-y)
Supplement: Supplementary file 5 — Additional file 5. Title: Results from all meta-analyses. Description: Results from all meta-analyses. [file 40337_2023_806_MOESM5_ESM.pdf]

| | Transgender men | | Transgender women | |
|---|---|---|---|---|
| Results from meta-analysis 1 (standardized mean difference, various instruments): | Vs. cisgender men | Vs. cisgender women | Vs. cisgender men | Vs. cisgender women |
| Main analysis (A and C) | 0.55 (0.26, 0.83) | 0.18 (-0.03, 0.40) | 0.37 (0.11, 0.62) | -0.25 (-0.79, 0.28) |
| Sensitivity analysis (A and D) | 0.84 (0.56, 1.12) | 0.30 (0.08, 0.52) | 0.56 (0.13, 0.99) | -0.51 (-1.36, 0.33) |
| Sensitivity analysis (B and C) | 0.54 (0.46, 0.62) | 0.16 (0.10, 0.23) | 0.36 (0.26, 0.45) | -0.15 (-0.23, -0.06) |
| Sensitivity analysis (B and D) | 0.76 (0.67, 0.84) | 0.24 (0.18, 0.31) | 0.47 (0.37, 0.57) | -0.20 (-0.28, -0.11) |
| Without possible outliers (A and C) | 0.46 (0.23, 0.70) | 0.09 (-0.05, 0.22) | 0.45 (0.22, 0.69) | 0.01 (-0.40, 0.42) |

| | Transgender men | | Transgender women | |
|---|---|---|---|---|
| Results from meta-analysis 2 (mean difference, only EDE-Q): | Vs. cisgender men | Vs. cisgender women | Vs. cisgender men | Vs. cisgender women |
| Main analysis (A and C) | 0.66 (0.50, 0.81) | 0.20 (-0.31, 0.71) | 0.76 (0.56, 0.95) | 0.40 (-0.22, 1.02) |
| Sensitivity analysis (A and D) | 0.71 (0.58, 0.84) | 0.22 (-0.18, 0.63) | 0.85 (0.63, 1.08) | 0.43 (0.01, 0.85) |
| Sensitivity analysis (B and C) | 0.66 (0.50, 0.81) | 0.22 (0.08, 0.37) | 0.76 (0.56, 0.95) | 0.47 (0.28, 0.66) |
| Sensitivity analysis (B and D) | 0.71 (0.58, 0.84) | 0.27 (0.14, 0.39) | 0.90 (0.76, 1.04) | 0.53 (0.39, 0.67) |
| Without possible outliers (A and C) | (no possible outliers excluded) | -0.10 (-0.29, 0.09) | (no possible outliers excluded) | (no possible outliers excluded) |

| Results from meta-analysis 3 (prevalence of ED (%)): | Transgender men | Transgender women | Overall |
|---|---|---|---|
| Main analysis (B) | 19.46 (17.72, 21.21) | 14.72 (12.45, 16.98) | 17.70 (16.32, 19.08) |
| Sensitivity analysis (A) | 20.18 (13.72, 26.65) | 18.26 (8.65, 27.88) | 19.30 (14.18, 24.43) |
| With studies reporting low prevalences (B) | 15.05 (12.76, 17.33) | 9.96 (7.24, 12.67) | 12.93 (11.18, 14.68) |
| With studies reporting high prevalences (B) | 25.57 (22.88, 28.26) | 25.68 (21.56, 29.79) | 25.60 (23.35, 27.85) |

A: random-effects model
B: fixed-effects model
C: upper bound on SD
D: lower bound on SD
