# Supplementary material for: Eating disorder symptomatology among transgender individuals: a systematic review and meta-analysis
Source: J Eat Disord. 2023 May 26;11:84. doi: 10.1186/s40337-023-00806-y (PMC10214585; doi:10.1186/s40337-023-00806-y)
Supplement: Supplementary file 6 — Additional file 6. Title: Outlier diagnostics from the meta-analyses. Description: Outlier diagnostics from the meta-analyses. [file 40337_2023_806_MOESM6_ESM.pdf]

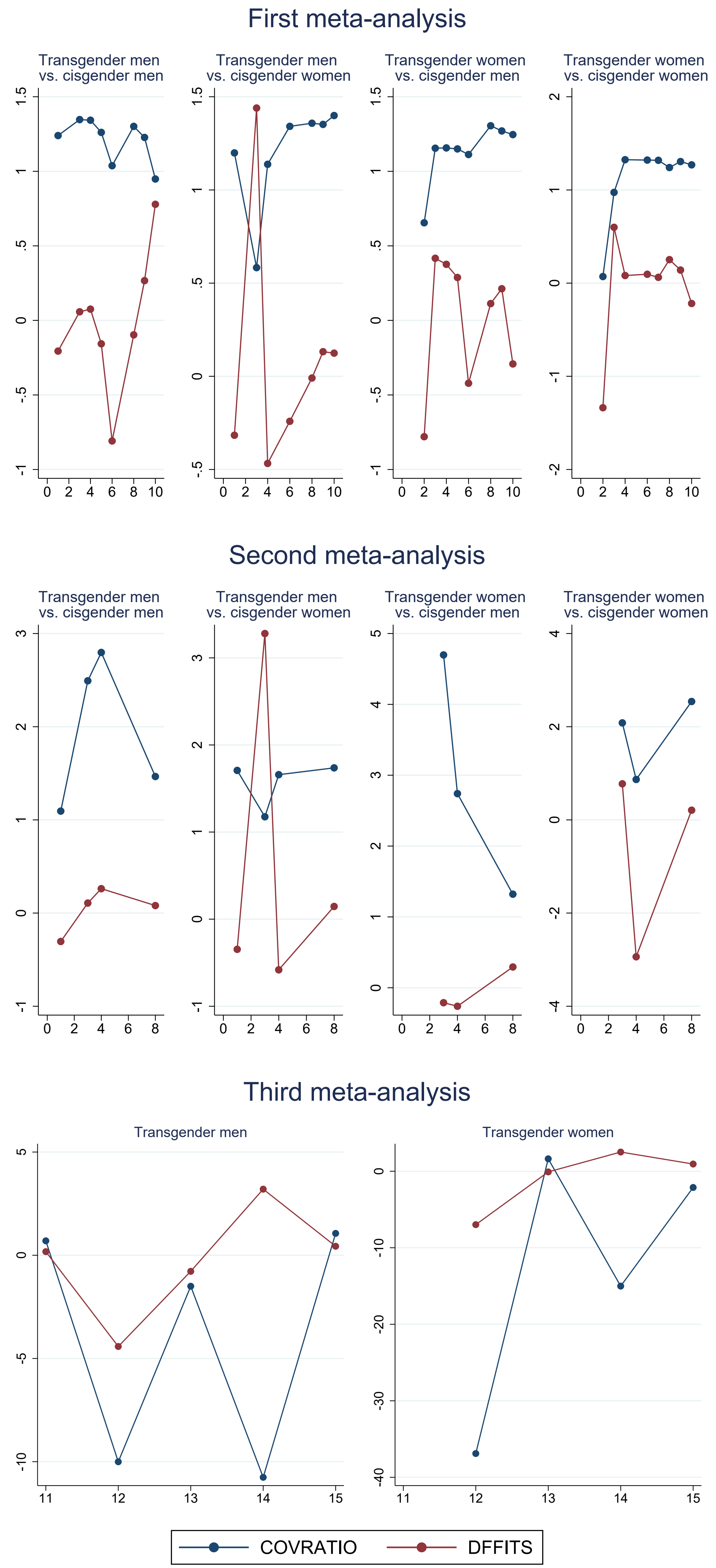

Note: The horizontal axes show the article ID
